# Chemical fingerprints of cold physical plasmas – an experimental and computational study using cysteine as tracer compound

**DOI:** 10.1038/s41598-018-25937-0

**Published:** 2018-05-16

**Authors:** J.-W. Lackmann, K. Wende, C. Verlackt, J. Golda, J. Volzke, F. Kogelheide, J. Held, S. Bekeschus, A. Bogaerts, V. Schulz-von der Gathen, K. Stapelmann

**Affiliations:** 1 Biomedical Applications of Plasma Technology, Ruhr University Bochum, Universitätsstr 150, 44780 Bochum, Germany; 2 ZIK plasmatis, Leibniz-Institute for Plasma Science and Technology, Felix-Hausdorff-Str. 2, 17489 Greifswald, Germany; 3 PLASMANT, University of Antwerp, Universiteitsplein 1, 2610 Antwerp-Wilrijk, Belgium; 4 Experimental Physics II, Ruhr University Bochum, Universitätsstr 150, 44780 Bochum, Germany; 5 Department of Nuclear Engineering, Plasma for Life Sciences, North Carolina State University, Raleigh, NC 27695 USA

## Abstract

Reactive oxygen and nitrogen species released by cold physical plasma are being proposed as effectors in various clinical conditions connected to inflammatory processes. As these plasmas can be tailored in a wide range, models to compare and control their biochemical footprint are desired to infer on the molecular mechanisms underlying the observed effects and to enable the discrimination between different plasma sources. Here, an improved model to trace short-lived reactive species is presented. Using FTIR, high-resolution mass spectrometry, and molecular dynamics computational simulation, covalent modifications of cysteine treated with different plasmas were deciphered and the respective product pattern used to generate a fingerprint of each plasma source. Such, our experimental model allows a fast and reliable grading of the chemical potential of plasmas used for medical purposes. Major reaction products were identified to be cysteine sulfonic acid, cystine, and cysteine fragments. Less-abundant products, such as oxidized cystine derivatives or S-nitrosylated cysteines, were unique to different plasma sources or operating conditions. The data collected point at hydroxyl radicals, atomic O, and singlet oxygen as major contributing species that enable an impact on cellular thiol groups when applying cold plasma *in vitro* or *in vivo*.

## Introduction

Cold physical plasmas are under investigation in various research fields, such as waste disposal, surface modifications, and medical applications. The latter field is highly attractive as it was already shown that CAPs assist in wound healing^1^, in the treatment of skin related disorders^2^, and in cancer treatment^3^. Plasmas can be designed by various excitation schemes as desired, such as jets, dielectric barrier discharges (DBDs), and other setups^4^. In addition, numerous feed gases can be used, such as ambient air or noble gases with and without molecular gas admixtures. This variability of plasma sources allows choosing a suitable source for a specific application. However, it also impedes comparison of experimental data or clinical results from different plasma sources. In plasma medicine, this challenge escalates as the biological targets can also vary widely, ranging from *in vitro* experiments using cellular components^5^, pro- and eukaryotic cell cultures^6,7^, animal experiments^8^, and observational patient studies^9^. While some general effects in biological targets are described *e.g*. improvement of blood circulation^10^ and cell proliferation^11^, mechanistic investigations on a molecular level are still lacking.

So far, it is generally assumed that reactive oxygen and nitrogen species (RONS) are key players in atmospheric pressure plasmas^12^, with many different types present including nitrite (NO_2_^−^), nitric oxide (NO), hydrogen peroxide (H_2_O_2_), superoxide (O_2_^−^), hydroxyl radical (OH), peroxynitrite (ONOO^−^), and singlet oxygen (^1^O_2_). Depending on their chemical properties and spatial distribution, activation of mammalian redox signalling systems is possible^13^ and thus modulation of *e.g*. antioxidant response, inflammation control, and cell fate. Extensive research points at the interaction of plasma derived ROS/RNS with biological matter *in vitro*, *ex vivo*, and *in vivo* (summarized in^14^). For example, NO and its storage forms, such as NO_2_^−^, are discussed as effectors in wound healing stimulation by plasma treatment^15,16^. In redox control, sulphur containing amino acids are a crucial component and numerous redox switches base on the reversible oxidation of thiol moieties, *e.g*. in cysteine. A targeting of these chemical groups by plasma-derived species has been suggested^17,18^.

Plasma sources investigated for medical applications are frequently used to treat liquids, which are subsequently applied to the biological target (indirect treatment)^19^. These treatment modalities are investigated for applications in which a direct treatment of the area of interest is not realistically achievable, *e.g*. intraperitoneal cancer and certain metastasis^20,21^. When used directly upon a model system or *in vivo*, initially a similar process is triggered: the plasma encounters a liquid surface and the deposition of RONS occurs^22^. Subsequently, these species intercept and modulate cellular redox signaling^23,24^. Therefore, the chemical footprint of a plasma source is of interest for three reasons: (i) fundamental understanding of chemical mechanisms and the identification of species deposited, (ii) comparison and optimization of plasma sources, and (iii) estimation of their respective biological impact. While long-lived ROS (H_2_O_2_) and RNS (NO_2_^−^, NO_3_^−^) are frequently detected in higher µM to low mM range (details see^25^), other non-radical species like atomic O, ^1^O_2_, and O_3_ are hard to detect directly with electron spin resonance spectroscopy being one option. Yet, the available probes are not specific when several species are present simultaneously^26^. Accordingly, their presence may rather be deduced from chemical traces left behind, especially in complex liquids^27^. To overcome this, Kogelheide *et al*. proposed cysteine as a model compound to compare the impact of various plasma sources and/or treatment conditions in a fingerprint-like manner^28^. Cysteine has already been shown to be one of the most affected amino acid when treated by plasma^29^. Furthermore, the chemical reactivity of sulphur-containing groups seem to play a major role in the time-dependent degradation of the chemical reactivity of plasma-treated liquids due to its H_2_O_2_-consuming properties^30^. Understanding of the interaction of cysteine and plasma-generated species has therefore benefits for both direct and indirect applications. In this work, experimental evidence is combined with molecular dynamics (MD) simulations to investigate the chemical impact of two plasma jet sources. The argon-driven kINPen and the helium or argon-driven COST-jet were compared for their chemical impact on cysteine in relation to the working gas composition applied. For both sources, the gas phase physics^31,32^ and observations in the biological^33,34^ and medical^8,35^ context are well described. While the published data indicates the major relevance of RONS for the interaction of plasmas with biological systems, so far limited information is available regarding their deposition in liquid systems. Cysteine has a simple yet diverse structure, allowing various covalent modification to be traced by FTIR or high-resolution mass spectrometry^28^ while still being small enough to perform reactive simulations. MD simulations inferring on potential reaction products were included to address the complexity of the reaction pathways, especially concerning feed gas modulation using the molecular gases oxygen and/or nitrogen. Such simulations have already been successfully used to observe, *e.g*., lipid peroxidation-induced pore formation in membranes^36^. The combined approach is tested for its ability to create distinguished “fingerprints” for the two plasma sources and respective treatment settings, and, ultimately, to grade the potential biological impact of the two plasma sources using a simple chemical gauge.

## Results

### FTIR analysis reveals the impact of plasma derived species on cysteine in dependence of plasma source and working gas parameters

**Figure 1:**
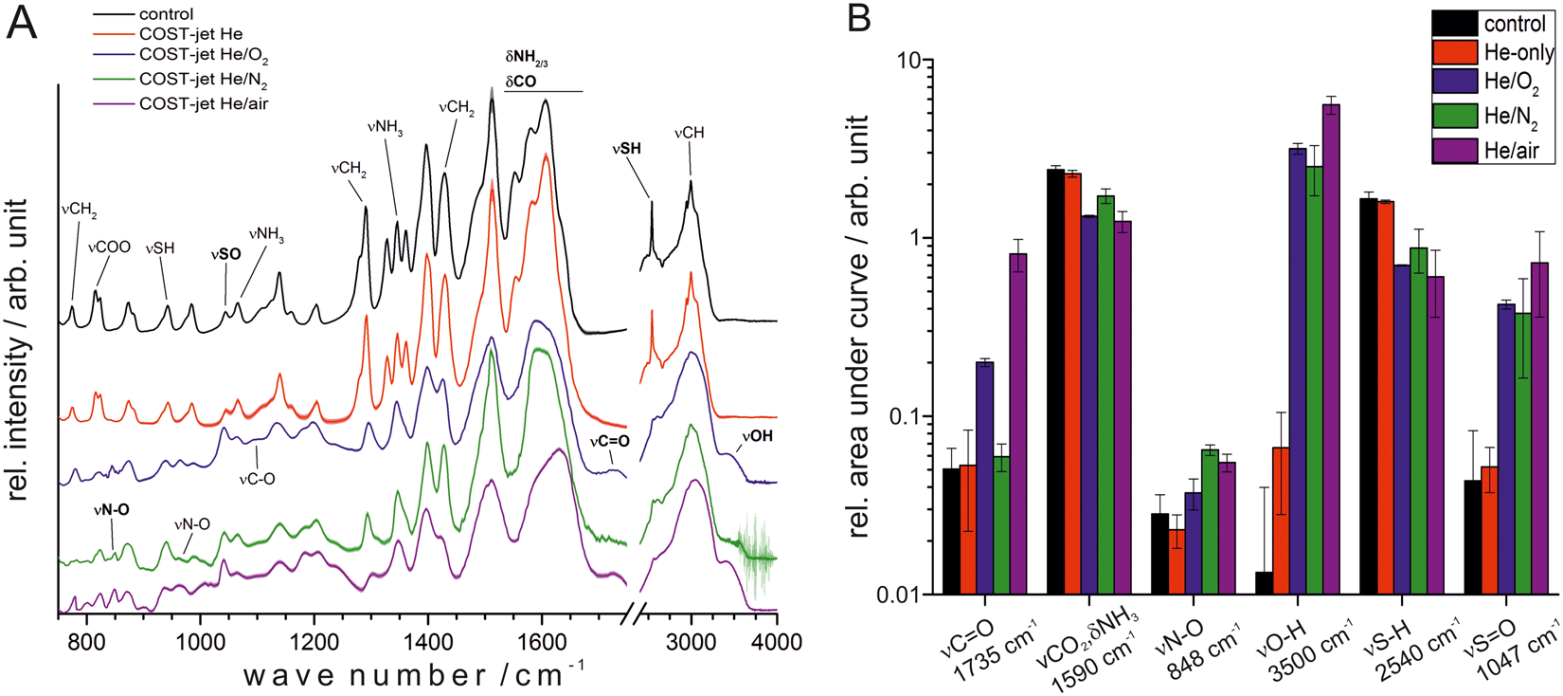
FTIR spectroscopy (**A**) and peak quantification (**B**) of He-based plasmas using the COST-Jet. Spectra were recorded after 10 min of treatment. Bold annotations were used for quantification. “Control” samples were treated only with He-only gas. The graphs are stacked for better visibility.

**Figure 2:**
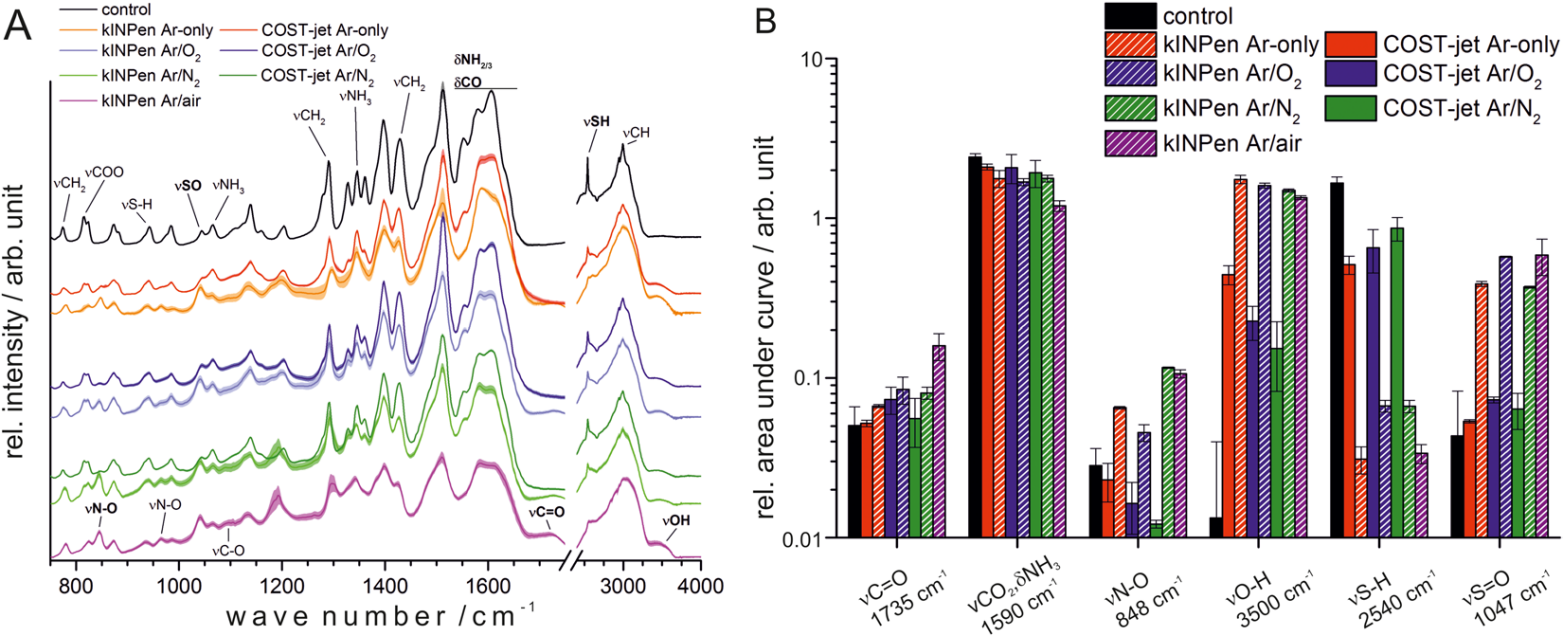
FTIR spectroscopy (**A**) and peak quantification (**B**) of Ar-based plasmas using both sources. Spectra were recorded after 10 min of treatment. Bold annotations were used for quantification. “Control” samples were treated with Ar gas only. The graphs are stacked for better visibility.

No macroscopically visible changes to the liquid was observed after cold physical plasma treatment and FTIR spectroscopy was performed to gain an overview of the plasma’s chemical impact on the cysteine model. Using the COST-jet with He-based plasma, only slight changes on the FTIR trace in comparison to control were found (Fig. 1A). A In contrast, small molecular gas admixtures rendered the plasma chemistry to effectively impact cysteine, modulating the presence of several chemical groups (Fig. 1A). In case of the Ar-based kINPen plasma jet, a similar but less pronounced observation was made with Ar-only plasma having a lower impact on the cysteine chemistry than Ar with molecular gas admixtures (Fig. 2A). The Ar-based COST-jet showed a generally reduced impact on the cysteine when compared to the kINPen but also to its He-based counterpart (Fig. 2A). Overall, certain differences between the plasma sources and, in case of the COST-jet, feed gases, became apparent, though the general trend for both sources was comparable for similar feed gas compositions. Basing upon results published previously^28^, attention was focused on the free thiol group of cysteine. Loss of the ν(S-H) signals at 940 cm^−1^ and 2540 cm^−1^ could be observed together with an even stronger increase of the ν(S = O) signal at 1040 cm^−1^. All cases using the kINPen with Ar/O_2_ or Ar/air resulted in a significant intensity increase of ν(S = O) and, vice versa, in a strong loss of the ν(SH) signal. The ν(OH) signal (3470 cm^−1^) followed the trends of the sulphur moieties and was found increased especially with molecular gas admixture (COST-jet He). A rise of the ν(OH) signal by two orders of magnitude occurred in all cases of He plasmas with admixtures, indicating strong oxidative processes (Fig. 1B). In opposition, the Ar-based COST-jet introduced this group more efficiently than the supplemented counterparts. For kINPen, comparable ν(OH) signals were observed for all gas variants used (Fig. 2B). NO bound signals (848 cm^−1^ and 964 cm^−1^) decreased upon Ar-based COST-jet treatment whereas they increased after kINPen treatment. Interestingly, the N-O signal was significantly more intense using an Ar-only or Ar/N_2_ plasma compared to Ar/O_2_ plasma. However, He-based treatment with the COST-Jet also showed slight increases of ν(NO), especially when using He/N_2_. Other signals of oxygen-containing groups also increased with the most pronounced signal being the doubling of the ν(C = O) band (1735 cm^−1^) when applying a He/O_2_ plasma, which increased further to about fourfold in case of a He/air plasma (COST jet, Fig. 1B). In contrast, only slight increases were observed when using Ar-based plasmas (Fig. 2B). For all treatment conditions, except for the He-only plasma, a certain impact on the cysteine backbone structure could be observed. The signals between 1300 cm^−1^ and 1650 cm^−1^ (the amino, carboxyl, and α-C regions) as well as the band around 3000 cm^−1^ (ν(C-H)) were sharp and defined in the untreated control and became less defined after plasma treatment. These changes were most pronounced after He/O_2_ treatment with the COST-jet as well as Ar-only or Ar/air treatment with the kINPen.

### High-resolution mass spectrometry reveals that molecular pattern of covalent cysteine modifications can be used to differentiate between plasma sources and process parameters

**Figure 3:**
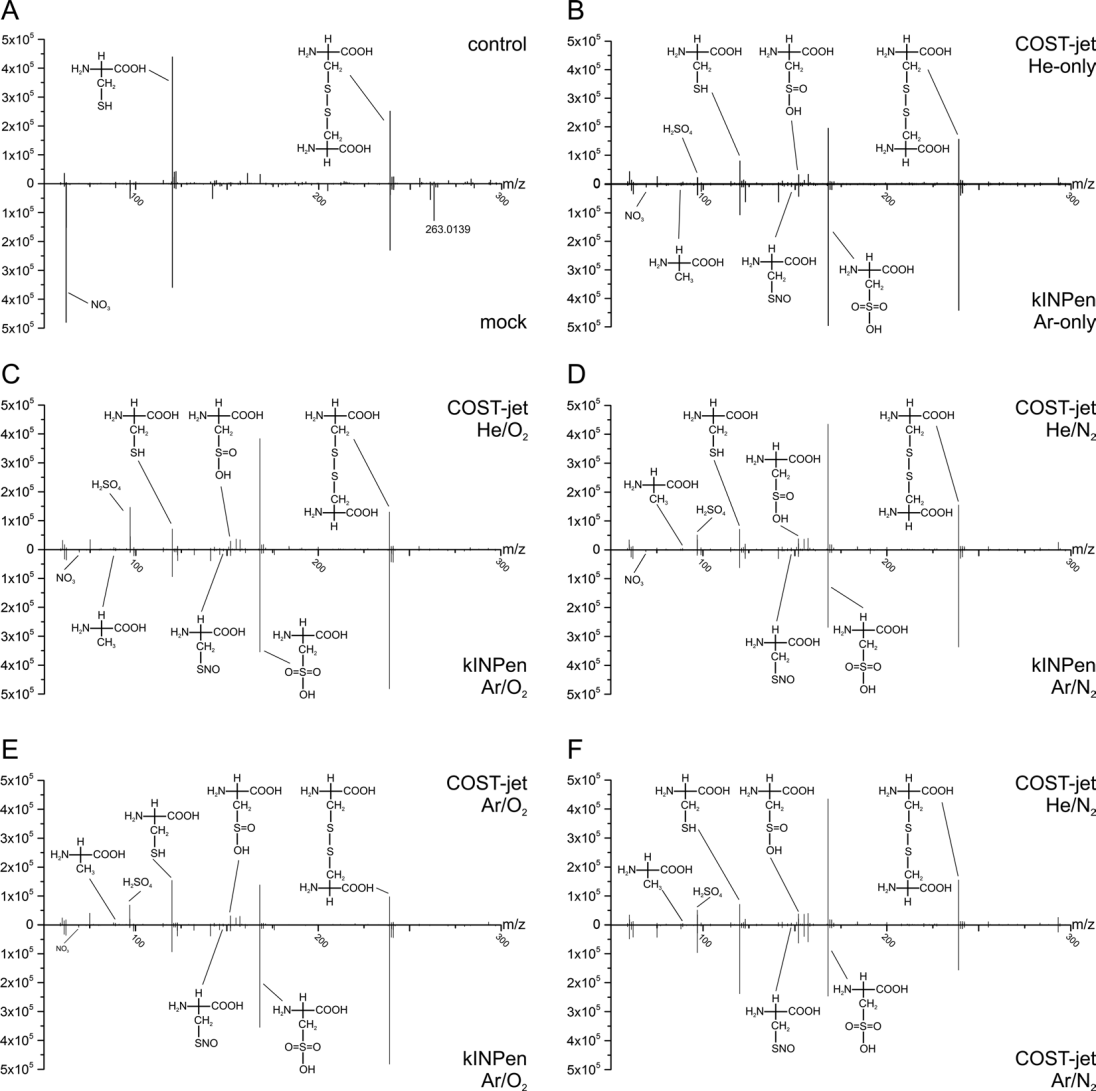
Mass spectrometry mirror plots of plasma-treated cysteine. Survey mass spectra of cysteine solutions, treated for 10 min with COST-jet or kINPen using varying working gas compositions. “Control” was treated with Ar-only gas (kINPen) for 10 min. “Mock” indicates the combined incubation of cysteine solution with 108 µM H_2_O_2_, 200 µM NO_2_, and 350 µM NO_3_ to simulate impact of long-lived plasma-generated species.

**Figure 4:**
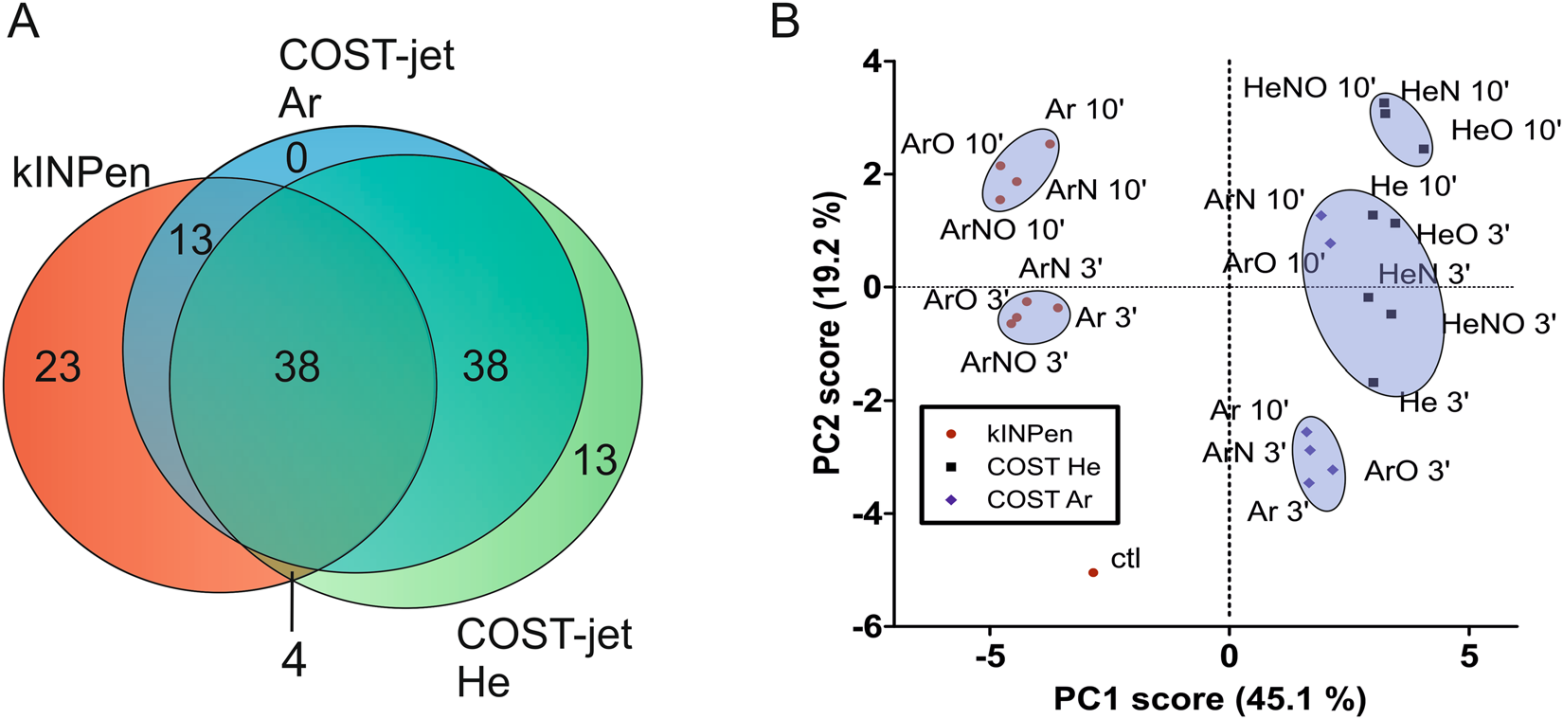
Venn diagram of all detected cysteine modifications (**A**) and corresponding PCA (**B**). Overall, 129 significant products were detected specific to the shown conditions (**A**). The percentages for the principal components (PC) indicate how much spectral differences can be described with PC1 or PC2, respectively.

To refine the analysis of cysteine modifications, sensitive high-resolution MS was used. Survey spectra as well as corresponding fragmentation spectra were acquired, enabling an accurate determination of the chemical modifications induced by the treatment. Control samples showed a few signals only: Unmodified cysteine (120.0119 m/z) and the homo-dimer cystine (239.0160 m/z), along with minor impurities and in-source fragmentation products (Fig. 3A). The number of signals increased significantly after any plasma treatment, corresponding to various products evolved (Fig. 3) with the cysteine’s sulphur moiety being predominantly attacked. The overall product profile for all plasma treatment conditions showed a large overlap of shared products (38, Fig. 4A). Among these, bisulphate (96.9596 m/z), cysteine, sulfinic (152.0018 m/z, Cys-SO_2_H) and sulfonic acid (167.9967 m/z, Cys-SO_3_H), and cystine, were the most major signals. Except for Ar-COST jet, plasma sources led to the formation of unique products (kINPen 23 signals, He-COST jet 13 signals; Fig. 4a), emphasizing the models ability to discern plasma source and their respective parameters. In detail, a large number of different covalent modifications of cysteine was observed, especially when using molecular gas admixtures (N_2_ or O_2_) (Fig. 3C,D). Comparing the influence of the source itself, using similar feed gas mixtures (Fig. 3E) or comparing the influence of the feed gas (Fig. 3F), again indicated identical dominant reaction products, yet their intensities varied. While cysteine was consumed, the signal of cystine as one dominant oxidation product occurred at high intensities. Oxygen admixtures in both sources favoured the formation of Cys-SO_3_H while its chemical predecessor Cys-SO_2_H was favoured by less oxidizing treatment conditions. Due to its chemical instability, no traces of sulfenic acid (Cys-SOH, 136.0068 m/z) could be detected. S-nitroso cysteine (Cys-SNO, 149.0011 m/z) was found in small quantities when N_2_ or N_2_/O_2_ was added to the working gas. After 3 min of treatment, its abundance was higher for the COST-jet than for the kINPen. Further, elimination products were detected under strong oxidizing conditions, among them alanine (88.0392 m/z) and bisulphate. A minor signals could be attributed to a dehydrogenated cysteine (117.9951 m/z), such as a thion, imine, or a C-C unsaturated cysteine. Numerous further signals were found, but due to low abundance or non-significant fragmentation products, not all could be identified. In addition to cysteine-derived modifications, MS/MS experiments revealed cysteine derived products. Most notably, signals at 271.0058 m/z (C_6_H_11_N_2_O_6_S_2_), 302.9463 m/z (C_6_H_11_N_2_O_8_S_2_), and 336.9857 m/z (molecular formula not yet established) were detected with minor to mean intensities. In parallel, the cystine peak area decreased with treatment time. Control experiments using cystine instead of cysteine as target molecule revealed its direct attack by plasma derived reactive species (data not shown).

### Mock treatment by emulating long-living plasma-generated species

To compare the effect of plasma treatment to the impact of long-living species present in the liquid bulk, *e.g*. H_2_O_2_, NO_2_^−^, and NO_3_^−^, concentrations of these three species were determined for all plasma conditions after 10 min of treatment (see Fig. S1). The pH was observed for its significant impact on reaction rates of thiol groups and found grossly unchanged. To generalize the results from the mock treatment and acknowledging the known chemistry of these species^25^.

Cysteine was either incubated with 113.5 µM H_2_O_2_, 200 µM NO_2_^−^, or 350 µM NO_3_^−^ at the lowest observed pH (5.8) or by a combination of all three. Treatment with H_2_O_2_ alone or in the combination showed little effect except a slightly increased cystine level. Addition of NO_2_^−^ or NO_3_^−^ alone or in the combination yielded no significant products. A strong signal at 263.0139 m/z was attributed by its isotope pattern and MS/MS spectrum to iron contamination of the used nitrite salt forming a ferric formiate cysteine cluster.

### Fingerprinting using principal component analysis shows distinctive product pattern

While the majority of the products was found frequently, some products were only present under specific conditions, (Fig. 4A). Twenty three (23) unique peaks were detected in kINPen-treated samples and 13 unique peaks were found using He-fed COST-jet. In contrast, no unique products occurred after COST-jet treatment using Ar working gas. Ar-based plasmas in both devices shared specific 13 products, while another 38 specific peaks were shared between the feed gases in the COST-jet (full peak list in supporting information). To compress the large data set and allow an easier comparison of the plasma sources and treatment conditions in an unbiased way, a “fingerprint” for each treatment condition was created using principal component analysis (PCA, (Fig. 4B)). PCA allows the reduction of large sets of dependent variables (*e.g*. full MS survey spectra) to a small data set of independent variables while retaining most of the information of the large set. Each PC accounts for as much variability in the data as possible by different loadings. PC1 has a strong negative loading of the cystine signal, meaning a high cystine signal pushes the PC1 score of a data set into the negative. In contrast, PC2 is strongly influenced by a negative loading of cystine as well as a positive loading of Cys-SO_3_H. Using the two most relevant principal components (PC1 and PC2), clusters could be identified relating to specific conditions. The PCA emphasized the differences between kINPen (red dots) and COST-jet (black squares and blue diamonds for He and Ar, respectively), as well as, to a lesser extent, the impact of treatment time. As expected, 10 min treatment time generally had a larger impact on the product profile than 3 min. Spectra could be identified belonging to either plasma source using PC1. PC2 indicated that spectra could be differentiated between Ar-based and He-based feed gases, even within the same plasma source (COST jet).

### DFTB simulation of cysteine oxidation by RONS shows good correlation with experimental

**Figure 5:**
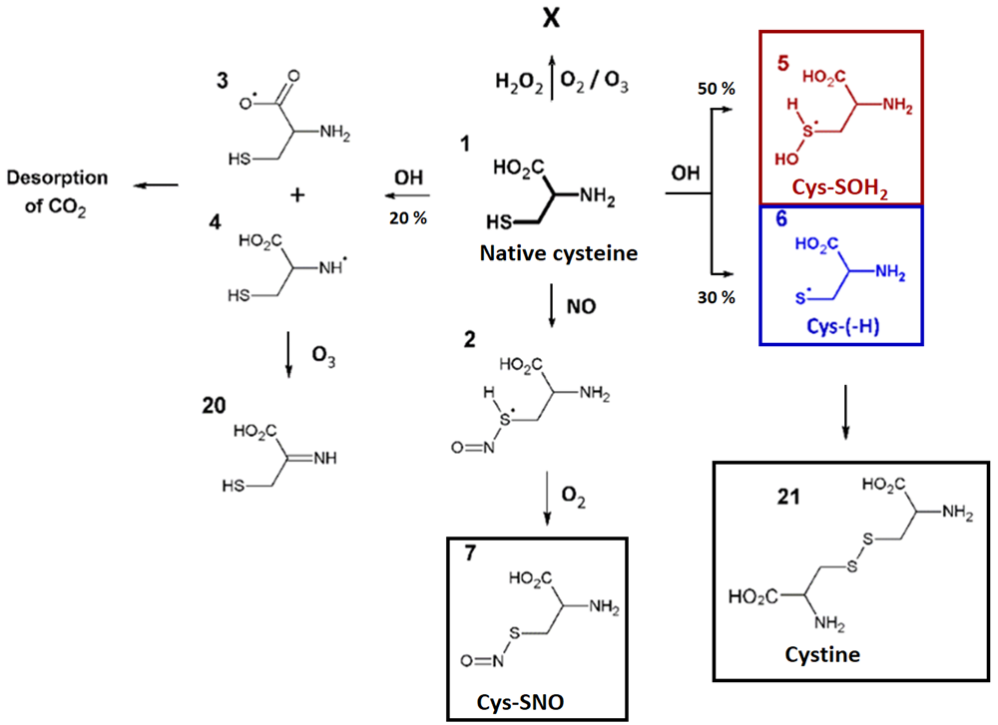
Summary of the predicted reaction paths encountered during the oxidation of native cysteine. A solid black box indicates stable oxidation products. Important intermediates are marked using dashed coloured boxes. As the interaction of cysteine with OH radicals resulted in multiple reaction products, branching ratios are depicted on the figure (based on the total number of reactions observed).

**Figure 6:**
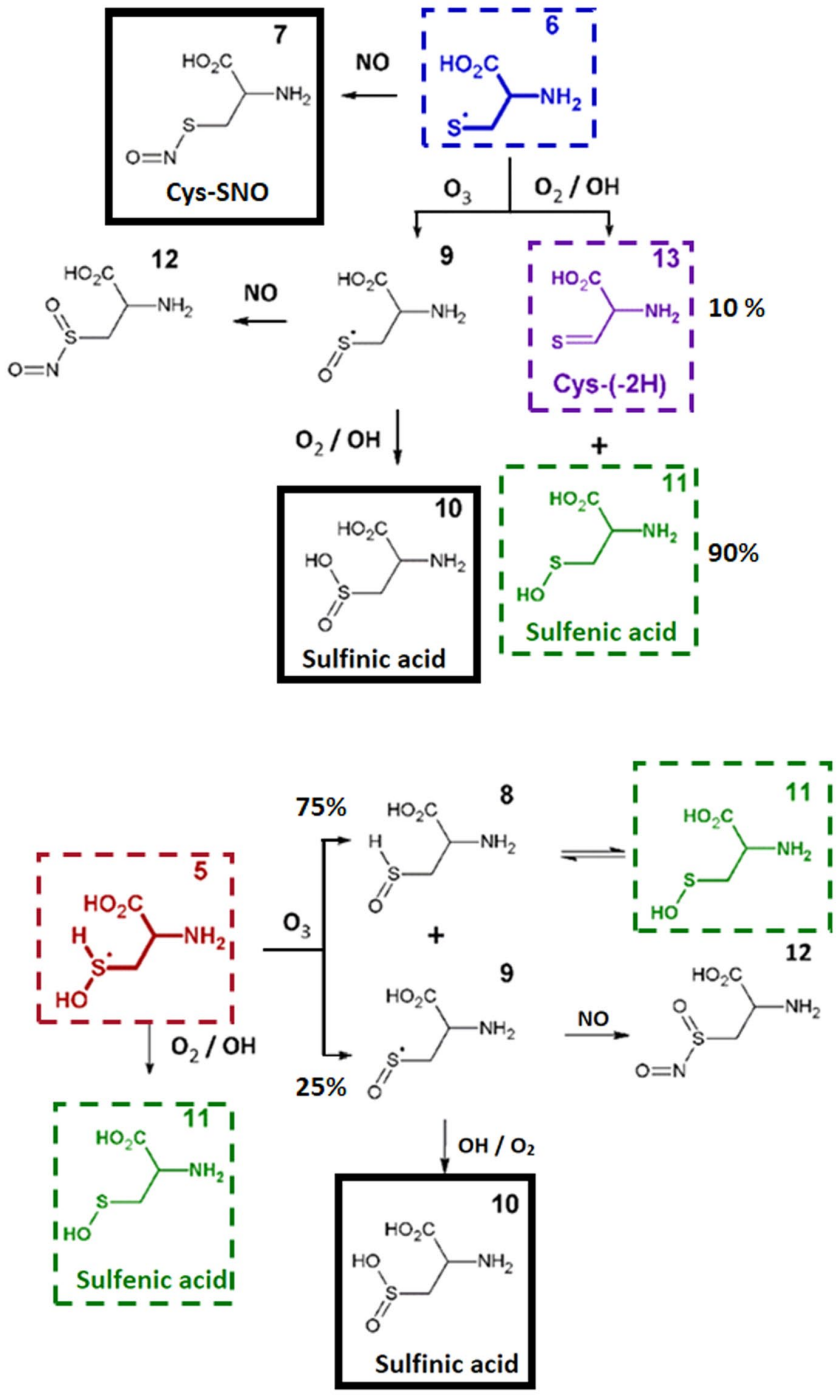
Summary of the predicted reaction paths encountered during the further oxidation of Cys-(-H) (6; top) and Cys-SOH_2_ (5; bottom). Important oxidation products are indicated using a solid black box. Important intermediates are marked with dashed coloured boxes. Branching ratios (compared to the total number of observed reactions) are depicted in cases when multiple reactions products were observed during the simulations.

Computational analysis using DFTB was used to infer on the cysteine products expected after plasma treatment. Figure 5 provides an overview of the simulated reactions, together with the most frequently observed products during these calculations. Over the course of 10 ps, oxidation of cysteine was initiated by a reaction with either OH or NO radicals, while only weak interactions were encountered between cysteine and H_2_O_2_, O_2_ or O_3_. It should be noted that reactions with these species might still occur after significantly longer timescales. Indeed, reactions with long-lived species, such as H_2_O_2_, are known to occur on cysteine as well. However, this requires certain conditions. Indeed, H_2_O_2_ reacts via a nucleophilic substitution on deprotonated cysteine, favoring basic solutions^37,38^. This can be expected to become important in the bulk of the solution over longer time-scales. The simulated reactions with OH and NO radicals resulted in the formation of cysteine adduct radicals, products (**2**) to (**6**). Here, Oxidation was mainly initiated on the thiol group, where the abstraction of the H-atom resulted in the formation of Cys-(-H) (**6**), while Cys-SOH_2_ (**5**) and Cys-SNO (**7**) were formed after an additional reaction of OH and NO, respectively. In the case of the OH radicals, the addition reaction was preferred over the H-abstraction: in 50% of the observed reactions, OH was found to add on the molecules, while H-abstraction reactions were only found in 30% of the total number of reactions. Reactions with OH on the cysteine backbone lead to the formation of products (**3**) and (**4**), albeit at significantly lower numbers (<5% of the total OH impact simulations). The latter reactions were usually followed by an interaction with O_2_ and O_3_ leading to desorption of CO_2_ and the formation of an imine group (**20**). The observed adduct radicals (**2**), (**5**), and (**6**) could react further with various reactive species. This propagation was investigated and created a plethora of reaction pathways, depending on the introduced reactive species. In case of radical (**2**), the oxidation was terminated through an H-abstraction reaction, forming Cys-SNO (**7**). Based on the experimental data, follow-up reactions of Cys-SOH_2_ and Cys-(-H) were investigated in more detail (Fig. 6). The calculations indicated that both adduct radicals could react with OH radicals, O_2_, and O_3_. When interacting with O_3_, the formation of radical (**9**) was observed which, in turn, formed Cys-SO_2_H (**10**) in the presence of OH or O_2_. Furthermore, when O_2_ or OH interacted with Cys-(-H), an addition or H-abstraction reaction was encountered, forming Cys-SOH (**11**), or an S = C double bond (**13**) with 90% and 10% of the total number of reactions observed, respectively (accounting for 88% and 6% of the total number of performed simulations). The formation of Cys-SOH was also encountered after the reaction of product (**5**) with O_3_. Here, next to product (**9**) leading to the formation of Cys-SO_2_H, product (**8**) was formed which is a tautomer of Cys-SOH^39^ (in 25% and 75% of the total number of reactions observed, respectively). As Cys-SOH is greatly favoured over its tautomer in solution, the former could be expected in solution after reaction with O_3_. Furthermore, the addition of NO onto Cys-(H) was encountered, which yielded Cys-SNO (**7**), like the interaction with native cysteine. Besides interactions with reactive species, the formation of disulphide bonds was investigated. For this purpose, the interaction of Cys-(-H) with either native cysteine or with another Cys-(-H) adduct radical was simulated. The simulations indicated that cystine (product (**21**), Fig. 5) would be formed almost immediately when two Cys-(-H) radicals interact with each other in solution, while an additional H-abstraction is required during the interaction with native cysteine.

**Figure 7:**
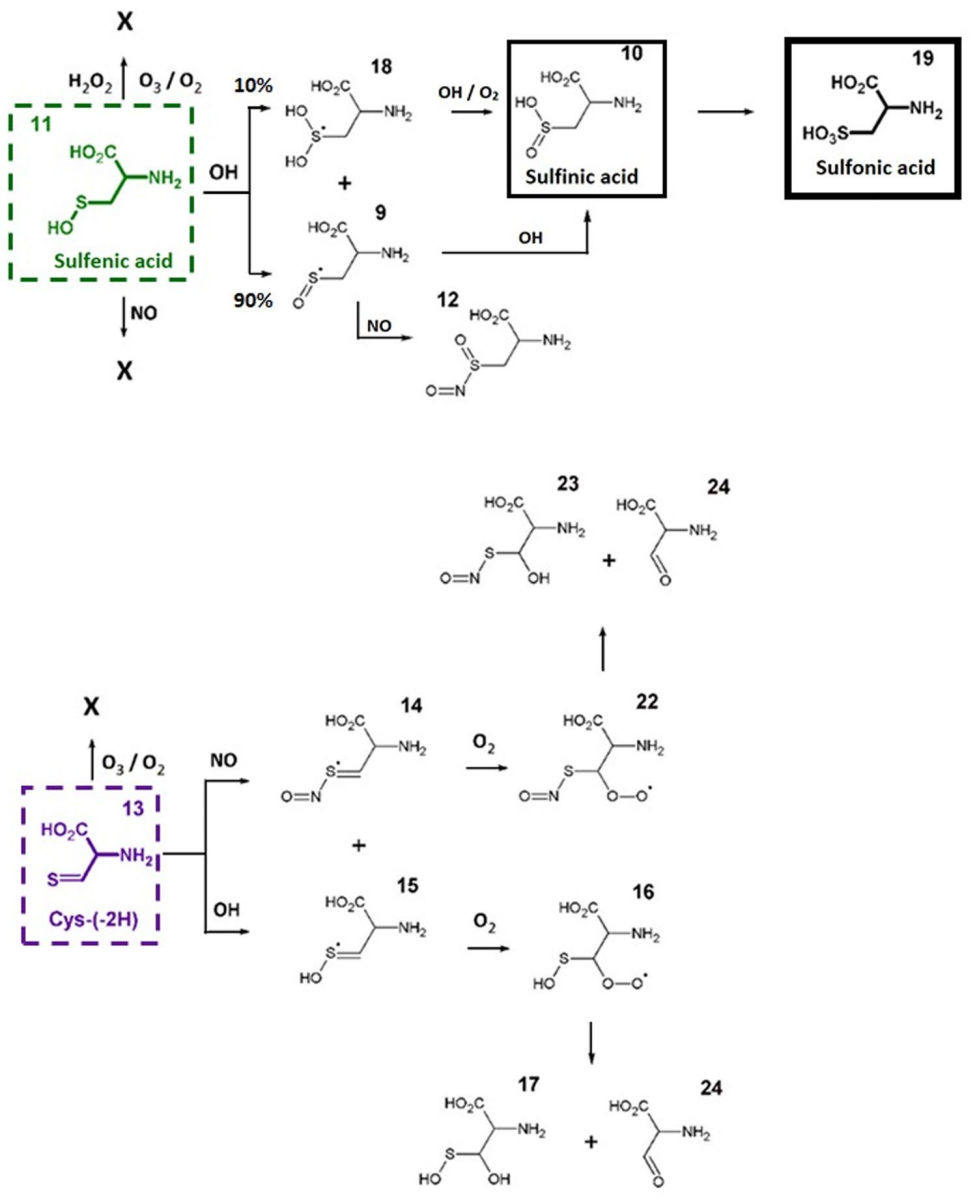
Summary of the predicted reaction paths encountered during the oxidation of sulfenic acid (top) and Cys-(-2H) (bottom). Stable oxidation products (sulfinic and sulfonic acid) are indicated using a solid black box. Branching ratios (compared to the total number of observed reactions) are depicted in cases when multiple reactions products were observed during the simulations.

Finally, Cys-SOH (**11**) and Cys-SO_2_H (**10**) as well as Cys-(2H) (**13**) were considered for further oxidation (Fig. 7). In the case of Cys-SO_2_H, the initiation of oxidation was only observed when interacting with OH radicals. Results were either an H-abstraction reaction, leading to the formation of product (**9**) and therefore to the formation of Cys-SO_2_H in aerobic conditions, or an OH addition reaction leading to the radical (**18**) which can react with OH or O_2_ forming Cys-SO_2_H. Following this reaction, the formation of Cys-SO_3_H (**19**) from Cys-SO_2_H by OH and O_2_ can also be expected. Finally, Cys-(-2H) (**13**) was found to react with both OH and NO radicals. The calculations indicated that products (**14**) and (**15**) form peroxides (ROO•) in aerobic conditions. It is known from literature that ROO•can react further forming both alcohol and carboxyl groups^40^. These last steps were not simulated, as this would require more complex systems as well as longer time-scales, which are beyond the limitations of the used computational method. However, considering this knowledge, the formation of products (**17**) and (**23**)/(**24**) from the observed peroxides could be expected. Note that product (**24**) is the result of desorption of the oxidized thiol group.

## Disscussion

Cold physical plasmas are of interest for the treatment of various medical conditions. While the mechanisms of action is not fully understood, redox related signalling plays an integral part: all plasma source produce a number of reactive oxygen and nitrogen species relevant for signalling processes *in vivo*. Density and type of species can be tailored by plasma source engineering, yet the ultimate impact on biological systems is rarely revealed. Typically, characterization of a cold physical plasma source focusses on physical aspects, such as electron density, electric fields, or gas phase species distribution and their respective chemistry. As such, both plasma sources investigated in this study are well characterized in the gas phase^41,42^ and to a limited extent in the liquid phase^43,44^. While these investigations allow deep insight into the gas phase chemistry of cold plasmas, they only offer a limited idea how plasma derived reactive species affect a biological molecule as the downstream reactions occurring in the liquid phase in interaction with the gas phase are complex. As an example, Sakiyama *et al*. presented a simulation of a micro discharge in contact with a liquid surface^45^. While the plasma source differs, various RONS, which are also produced by the sources investigated in this work, were covered by the simulation resulting in 624 reactions, many in dependence to each other. This high complexity indicates the need for better and easy-to-use models at the end of the reaction scheme (=the treated target). To circumvent the extra complexity of a large biomolecule or intact cells/organelles, small target molecules can act as sensor systems for the plasma triggered liquid chemistry. In this regard, some studies with other small molecules, such as tyrosine and phenol, are available for the kINPen (tyrosine)^27^ as well as for the COST-jet predecessor (the µAPPJ, phenol)^22^. It was apparent that both sources caused chemical modifications on these molecules, though the scope in both cases was focused on a few specific modifications, accentuating the need of an extended approach monitoring aiming to monitor all occurring modifications.

The kINPen and the COST jet, the two plasma sources used, differ in driving power frequency (1 MHz vs. 13 MHz) and electrical field (parallel or perpendicular to the effluent, see Experimental). Accordingly, reactive species output and supposed impact on the tracer molecule are different. Using FTIR spectroscopy, a significant impact of either plasma source on cysteine was revealed, with differences being smaller than expected from the distinct physical parameters of the plasma sources. The reactivity of the SH group dominated the product profile with an emphasis on the presence of S=O signals and a subsequent loss of SH signals, correlating with treatment time and molecular gas admixtures. The parallel increase of OH groups observed can be explained by the stepwise oxidation of thiol groups and is backed by the alongside loss of the ν(SH) signal. Yet, the comparable strong signal indicates that other residues might be carrying OH-groups as well. Additionally, changes in the spectra (δ(NH_2_), δ(C=O)) indicated rearrangement in the carbon backbone with potential reaction pathways leading to aldehydes or ketones, addition-elimination reactions, or the oxidation of the carboxyl group, resulting in its loss as CO_2_, (MD simulations, Fig. 5). Overall, the extent appears rather small compared to the intensity changes related to the SH group, demonstrating that the general structure of the cysteine remains predominantly intact. This indicates that under the given conditions, cold plasma derived-species do not indifferently attack organic molecules until their mineralization (CO_2_/H_2_O) but facilitate covalent modification.

To augment data obtained from FTIR, high-resolution mass spectrometry and subsequent MS/MS approaches were applied. In general, mass spectrometry revealed that in contrast to a dielectric barrier discharge plasma^28^, all dominant modifications were observed around the thiol moiety, albeit with significantly different quantities. Treatment time affected product formation though not in a strictly linear fashion (Fig. 3 and supporting files), as intermediate products can be utilized by the plasma generated species (see results part). Such, 128 different signals with an intensity >1000 counts s^−1^ where detected. Using statistical tools, distinct clusters of samples could be defined, consisting of samples treated with kINPen (PC1 score < −2) or COST jet (PC1 score > 1) (see Fig. 4). These differences could be contributed dominantly to cystine (m/z 239.02) and nitrate (NO^3−^, m/z 61.99, lowest PC1 values), m/z 293.18 (unassigned), m/z 155.00 (unassigned), bisulphate (m/z 96.96, highest PC1 values), sulfonic acid (m/z 168.00, highest PC2 value), and cysteine (m/z 120.01, lowest PC2 value; complete data see supplementary information). This reflects the plasma conditions: presence of N_2_, O_2_, or the mix of both in the working gas and long treatment times favour sulfonic acid, leading to PC2 scores > 0 (quadrants 1 and 4). Under these conditions, atomic O, N, or various RNS are abundant at the gas – liquid interface. PC2 scores < 0 can be explained by a less effective plasma treatment (Ar-based COST jet, short treatment times). Low PC1 scores, as found for the kINPen treated samples are indicative for high cystine content of the samples, which derive from H_2_O_2_ deposited by this plasma source. Further, stable nitrate proclaims the intermediate presence of short-lived nitrogen species, caused by the kINPen’s effluent significant interaction with the ambient air facilitating RNS production^46^. In contrast, the COST-jet’s plasma displays a 1 mm effluent, preventing strong interaction with the ambient. The chemistry of the contributors to the high PC1 scores found for the COST jet are not known so far, except for bisulphate, which indicates active oxidation and subsequent elimination reactions with bisulphate and alanine as fragments. It might be possible that they are elimination products stemming from the breakdown of Cys-SO_3_H but its accumulation even after long treatment times speaks against it. Cys-SO_2_H and mixed cystine-S-sulfoxides or fragments thereof might be potential precursors. Their abundance inversely correlated with the two elimination products, as does the presence of cystine. Interestingly, the presence of highly oxidized products is seemingly in contrast to the atomic oxygen levels detected: measured with the same diagnostics higher and further reaching atomic oxygen densities were detected for the kINPen than for the COST jet^47,48^. Differences in gas flow mechanics are deemed responsible for this contradiction. Beside, numerous cysteine and cystine derivatives with lower abundances were detected, either due to instability or due to formation with low reaction rates. While many of them impede complete structural elucidation due to calculated masses higher than 290 and/or low MS/MS ion intensity, these compounds seem to be key in the differentiation of plasma sources or their parameters. The disulphide structure of cystine can further be targeted by reactive species, leading to the formation of mixed cystine-S-sulfoxides. Also, their oxidized disulphide structures carry biochemical activity, making these compounds potential mediators of the indirect plasma application^49,50^. According to literature, Cys-(S-S=O)-Cys evolves from the reaction of two cysteine molecules with ^1^O_2_ via a zwitterion (Cys-S + OO^−^), indicating its presence in the liquid after plasma treatment^51^.

Surprisingly few products detected by MS carry OH groups, contrasting the pronounced ν(OH) signal in FTIR. If this is due to the instability of the products in the electrospray ionization source or an overestimation of this chemical group in FTIR remains unclear. In principle, plasma generated OH radicals can abstract H atoms from the cysteine (at C2 and C3), and the resulting radical may recombine with another OH radical forming an additional hydroxyl group, fostering the breakage of the C-S bond under formation of an aldehyde via creating a thioacetal. However, given its instability, no such ion was detected by MS and *in situ* elimination reactions seem to be likely. Interestingly, only a slight impact on cysteine was observed by FTIR spectroscopy after treatment with He-only plasma (COST-jet): while FTIR spectroscopy only revealed very slight changes in the spectrum, significant Cys-SO_x_H signals could be observed by MS, indicating a high oxidative potential. Another explanation for the observed oxidative modifications might be that atomic oxygen is produced either by the interaction of plasma-generated VUV radiation with the ambient air and/or by small amounts of water impurities in the discharge itself. It is described that a He-only plasma can affect organisms by its strong UV and VUV radiation^32^, the latter reaching the sample by means of a transparent noble gas channel formed by the effluent^52^. Cysteine may be oxidized by intense UV radiation via the formation of ^1^O_2_^51^. Here, cysteine was treated in water, which prevents a strong direct (V)UV impact on the sample (penetration depth <0.05 mm).

**Figure 8:**
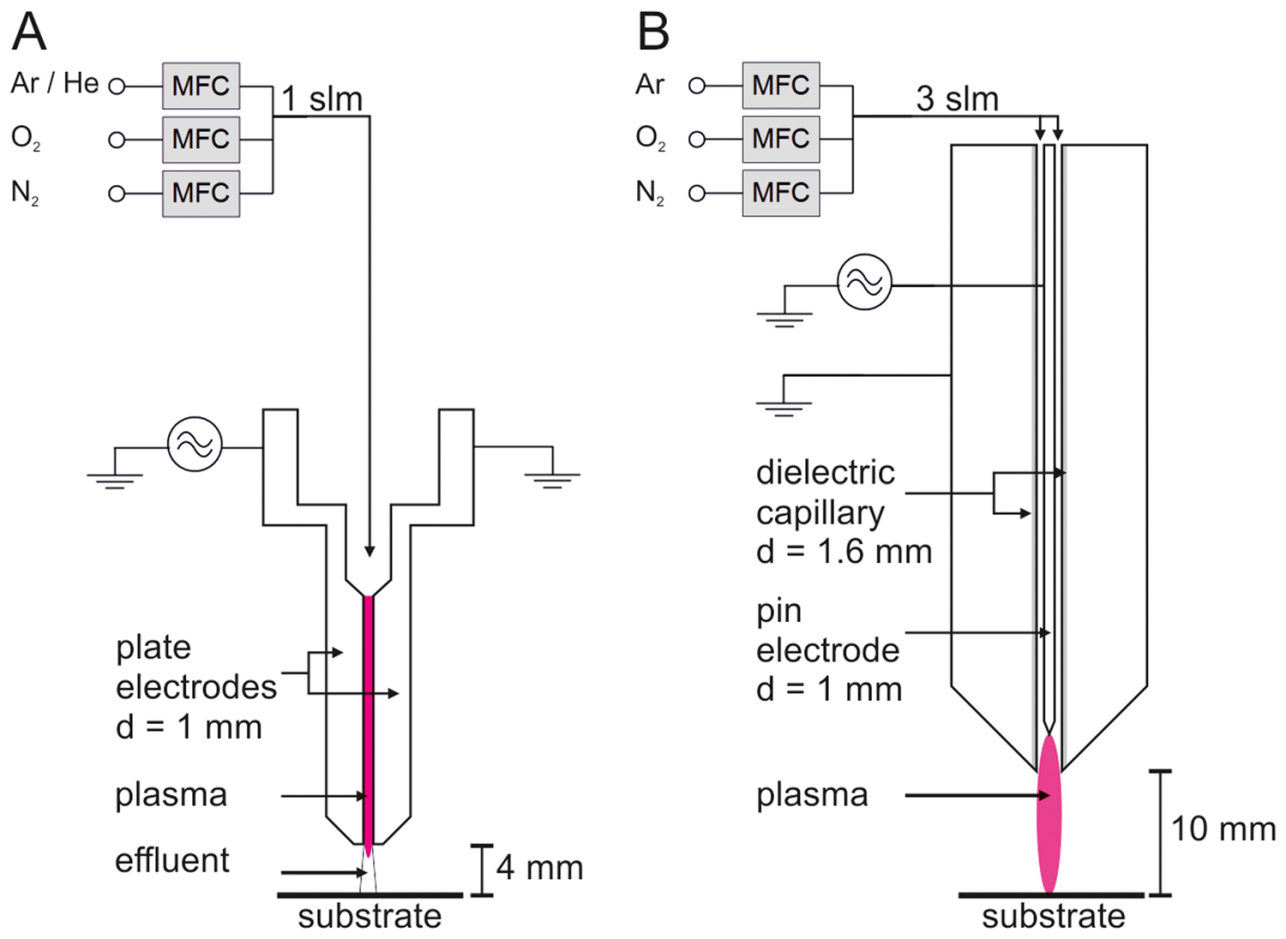
Schematics of the COST-jet (**A**) and kINPen (**B**). MFC: mass flow controller, slm: standard litre per minute.

In general, the two plasma jets at hand show some differences in their impact on the model substrate cysteine. The differences of the two devices are also illustrated by two publications on the atomic oxygen distribution in the effluent of the respective devices measured with the same diagnostics^47,48^. This difference in performance might be explained by the different excitation concepts. While the COST-jet’s plasma is a homogeneous high-pressure glow discharge, guided streamers develop in case of the kINPen^46^, with further implications on the production of RONS. In case of the COST-jet, the active plasma is confined to the electrode gap with an effluent of less than 1 mm length, preventing intrusion of ambient air into the discharge. Therefore, only long-living plasma-generated species can interact with ambient air in the effluent of the jet (Fig. 8) generating additional reactive species. In contrast, guided streamers from the kINPen protrude up to 10 mm into the atmosphere, capable of producing additional RONS while interacting with the surrounding atmosphere. Further differences occur by using different working gases (Ar or He) and admixtures (N_2_ and O_2_). The dissociation energy of O_2_ is about two times lower than for N_2_ (498 kJ/mol versus 945 kJ/mol). The stored energy in He metastables, being characteristic for atmospheric pressure He discharges, is at 20 eV (1929 kJ/mol), far beyond the dissociation levels of both molecules. Due to the lower dissociation energy of oxygen, the He discharge is particularly efficient as atomic oxygen source. In contrast, Ar metastable energy levels couple extremely well to molecular N_2_ states, leading to a more efficient dissociation of N_2_ by energy transfer. These differences might explain why the ratios between cystine and Cys-SO_3_H divert between the sources when *e.g*. oxygen is admixed. In case of the COST-jet, atomic oxygen is dominant, resulting in increased higher oxidation states (e.g. Cys-SO_3_H). In the kINPen, due to longer distance between active plasma core and treated liquid, atomic oxygen is present in lower abundances, replaced by the barely soluble O_3_ and subsequent less oxidized cysteine/cystine. Additionally, the larger amounts of RNS lead to stronger acidification of the liquid which causes a reduced reactivity of the SH moiety. While even slight changes in pH can have an impact on thiol reactivity^53^, besides the general slight acidic conditions due to the presence of cysteine, a drop of pH from initial 6.8 to about 5.8 was observed after treatment. This most likely stems from the fact that even though treatment time was relatively long, the treated volume was also quite large compared to other studies and treatment with jets affect the pH less than direct treatment with *e.g*. DBDs. An alternative kINPen source using a shielding gas setup^54^ has already been presented, though investigations regarding its chemical potential are still absent.

Product identification and result interpretation was fostered by computational analysis of cysteine modification by cold plasma derived species. Here, SCC-DFTB simulations of cysteine interacting with several reactive species generated by plasmas were performed. The reactive species considered were OH, H_2_O_2_, O_2_, O_3_, and NO in solution. ^1^O_2_, which is also a relevant species produced by various plasma sources, is not considered in this investigation as it cannot be distinguished from molecular oxygen in the ground state by the used method. In addition, atomic oxygen cannot be used as reaction partner in this model. Differences between experiments and simulations can also stem from significantly different time scales: 10 ps vs. minutes. As DFTB simulations are computationally demanding it is not possible to extend the simulation to seconds or even minutes. Nonetheless, the simulations expand the described cysteine model and allow for a more conscious annotation and a more in-depth interpretation of the induced modifications. The bilateral approach using simulation and experimental evidence improved product identification by fragmentation of precursors of interest during MS analysis (MS/MS) and in turn, experimental evidence proved indispensable for refining input variables for a second simulation step (*e.g*. product (**13**), Fig. 6).

While only stable reaction products can be observed by experimental means, simulations allow for a more in-depth view into short-lived reaction intermediates. In addition, they are highly useful for annotating MS and MS/MS signals. In due course, numerous potential reaction products have been identified by DFTB simulations (Figs 5–7). While experiments and simulations are in good agreement on major products, results differ in other cases, *e.g*. Cys-SOH (product (**11**), Fig. 6), which is calculated to be a primary product. Due to its instability under the experimental conditions, it was not detectable, a fate it shares with other potential candidates, such as (**11**), (**12**), and (**17**). In the case of Cys-SOH, it readily forms cystine by attacking the thiol group of cysteine^53^. Of the broad range of possible predicted modifications, only a selected number of structures could be observed in the experiments. Among those are cystine, Cys-SNO, and Cys-SO_3_H. Other molecules have instable chemical structures, *e.g*. (**5**) or (**17**), or are intermediate stages, *e.g*. (**16**) and (**22**), and accordingly hard or impossible to observe.

While our studies focused exclusively on an *in vitro* model, knowledge gained by these experiments will allow for a better understanding of *in vivo* effects of cysteine modifications. In both experiment and simulation, a major impact of the non-thermal plasma treatment on the thiol moiety was predicted or detected and the resulting S-S, S-O, or S-N bonds or double bonds can be relevant in inter- and intracellular redox signaling^55^. Cysteine is a major component of enzymes or signalling proteins and thiol modifications are used in many instances to regulate enzyme activity and function, *e.g*. via the inactivation of metal-sulphur clusters^56^, but can also cause significant issues if introduced unregulated, especially considering potentially irreversible modifications such as sulfonic acid^57,58^. Free cysteine is not stable, and easily oxidized to cystine, which is taken up into the cell by the glutamate cystine antiporter (SLC7/A11 or xCT, Q9UPY5). This transporter is under redox control (*e.g*. NRF2), improving the cells cysteine supply^59^. Especially cultured cells express the xCT system almost ubiquitously. It has an undetermined specificity, transporting the anionic form of cysteine, or glutamate, respectively. A neutral amino acid transporter system (alanine, serine, cysteine preferring; ASC) transports excess cysteine into the outside where it can be oxidized and taken up by the xCT again. This circle, which can reach a steady state in unchallenged situations, allows interference of plasma derived species with intracellular processes via cysteine/cystine couple. Oxidized cystine derivatives as detected after cold plasma treatment of cysteine might be transported into the cell if the carboxyl groups, which are relevant for the negative charge of the molecule, are unaltered by the treatment. Subsequently, cell either experience a lack of cysteine used for protein and glutathione synthesis or are able to reduce the oxidized disulphide bridge (*e.g*. product 271.01 m/z). Subsequently, redox related signalling, *e.g*. via NRF2 related cascades may be triggered as has been shown for plasma treated RPMI (containing 0.5 mM cystine) in human keratinocytes, leading ultimately to the protection of the cells^13^. As the extracellular cysteine/cystine pool is shared within a tissue, plasma related impact on cell physiology might be translated into deeper layers of the tissue that have not directly been in contact to the plasma or plasma treated liquid^19^. Especially in chronic inflammation, *e.g*. non-healing wounds, autoimmune diseases, the cellular environment is characterized by an oxidative potential, and the respective xCT system regulation so far not fully understood^60^. Such, the plasma can act as a tool to facilitate fundamental understanding in these conditions.

Besides purely oxidative modifications, nitrosated thiols were observed experimentally as well as in the simulation. Cys-SNO is discussed as a major NO donor in cells, *e.g*. during wound healing^15,61^, and is thought to be of regulatory impact in case of protein bound Cys-SNO^18^. From the point of application, an increase of Cys-SNO production would be beneficial in treating several disorders, especially chronic wounds. Here, the supply with NO is severely hampered and the topic application of species forming Cys-SNO desirable. As presented in the data, cysteine is efficiently oxidized by ROS, *e.g*. singlet oxygen, singlet oxygen (2.1 × 10^−7^ M^−1^s^−1^ at pH 7^62^), atomic oxygen (presumably fast, but no rates known), superoxide (around 10^2–3^ M^−1^s^−1^ as discussed in^63^), and to a small extend by H_2_O_2_ (about 14.7 × 10^−1^ M^−1^s^−1^ at pH 6^53^). In contrast, the formation of Cys-SNO, while relatively fast (2.6 × 10^−5^ M^−1^s^−1^ as shown in^64^) but is limited by the main nitrosating agent formation of N_2_O_3_ from NO and O_2_ is slow (6.6 × 10^6^ M^−2^ s^−1^)^65^, making its formation so far a minor contributor. A total reversion from oxidizing to nitrosative chemistry seems unlikely, but optimization of the feed gas composition and the surrounding atmosphere (curtain) might allow for an increase of Cys-SNO production. Future experiments will focus on understanding the balance between oxidative and nitrosative conditions.

The obtained data will serve as a starting point for those future investigations as it has already been shown that plasma can also interact with thiols in living organisms^66^. However, the far higher complexity of the full cellular environment will have to be addressed in the next step either by a more refined model or by the direct investigation of cells.

## Conclusions

The improved cysteine model allows both for an easy-to-use overview of the impact of plasma treatment on chemical groups using FTIR spectroscopy, as well as an in-depth investigation using MS-based PCA analysis. Our results show that plasmas cannot only be compared on the physical but also on the bio-chemical level in a relatively fast way. Interestingly, a few distinct chemical modifications proved to be dominant under all treatment conditions with other products forming reaction intermediates. These observations might lead to a new understanding of the interactions between biological targets and plasmas with a generalization of plasma treatment from a chemical point of view concerning its dominant modifications. However, more sources and conditions will have to be compared to explore the full range of possible modifications. A logical next step will be the correlation of observed modification patters with *in vivo* effects of plasma treatment. With a large enough library of modification patterns, gathered using different sources and parameters and correlated with *in vivo* data, the determination of main effectors of plasma-cell interactions might be possible. Furthermore, the low-abundance modifications indicate that a modulation of gas composition might offer an interesting way to focus on specific low-yield thiol modifications with potential medical benefits. Further studies based on observed modifications will be a reasonable first step to tune plasmas for specific and desired chemical modulation of cells, especially concerning thiol chemistry.

## Experimental

### Plasma sources and treatment conditions

The COST Reference Microplasma Jet (COST-jet, Fig. 8A) is the outcome of the European COST action MP 1011. It is capacitively coupled and driven by an AC voltage at 13.56 MHz. The feed gases used were either pure argon (Ar-only) or helium (He-only, both 1 slm), or the following mixtures: Ar/O_2_ (0.004% O_2_), Ar/N_2_ (0.01% N_2_), He/O_2_ (0.5% O_2_), He/N_2_ (0.5% N_2_), or He/air (0.1% O_2_ and 0.4% N_2_). All gases were of 5.0 purity. During treatment, the dissipated power was held constant at 590 mW for argon and 330 mW for helium. For details, see^67^. The kINPen plasma jet consists of a powered pin electrode (1 MHz, 2–6 kV_pp_, pulsed @ 2.5 kHz, details see^68^) shielded by a dielectric tube from the outer electrode (Fig. 8B). The dissipated power in pure Ar was around 500 mW. Three (3) slm Ar was used as the feed gas, either without any admixture or with O_2_ (1%, Ar/O_2_), N_2_ (1%, Ar/N_2_) or O_2_ and N_2_ (0.25% and 0.75%, Ar/air). For the experiments, the sources were mounted vertically above the treated liquid sample at a distance of 4 mm (COST-jet) or 10 mm (kINPen) in open atmosphere.

### Sample preparation

Cysteine (Sigma Aldrich) was dissolved in high purity deionized water to a final concentration of 100 µg/ml. Before and after treatment, samples were stored on wet ice. Samples of 3 ml were treated for 3 and 10 min for all conditions.

### FTIR spectroscopy

Cysteine samples were analysed and relatively quantified as described by Kogelheide *et al*.^28^. In short, 20 µl of each sample was desiccated on silicon wafers and 32 FTIR spectra acquired between 750 and 4000 cm^−1^ with a resolution of 4 cm^−1^. Ten (10) positions of each sample were measured using an FTIR micro spectrometer (Spotlight 200, Perkin Elmer). The measurements were done in triplicate. Afterwards, the samples were normalized and the areas-under-curve calculated for the chosen signal bands.

### Mass spectrometry

High-resolution MS data were collected using a TripleTOF5600 system with Analyst TF 1.51 software (both Sciex). The treated samples were diluted with 40% methanol containing 0.05% ammonia, and directly infused (10 µl/min) into the systems turbo ion source using negative electrospray ionization. Between 50 and 400 m/z were recorded and peaks were fragmented using CID. The system was calibrated daily using cysteine/cystine solutions. Data analysis was performed using Analyst TF 1.51, PeakView 1.1 (both Sciex), and Mass + +2.7.6 (Shimadzu).

### Emulating long-living reactive species generated by plasma

113.5 µM H_2_O_2_ (#H1009), 200 µM NaNO_2_ (#563218), and 350 µM NaNO_3_ (#229938, all Sigma Aldrich) were added to 3 ml of cysteine solution (pH adjusted to 5.8 with formic acid) either alone or all three species combined. After 10 min of incubation, reactions were stopped by freezing samples at −80 °C until measurement by MS (see above).

### Principal component analysis

Principal component analysis was performed using MarkerView 1.21 software (Sciex). The investigated data set contains all peaks detected with an ion flux ≥ 600 counts s^−1^ and the respective peak area. A total of 15 principal components (PCs) were identified by the software explaining between 45.1% and 0.6% of variations in the sample set. PC3 and following PCs only explained 9% or less of differences, therefor only PC1 and PC2 were plotted against each other. Logarithmic scaling and Pareto weighing was used to visualize the results. A Venn diagram was calculated using Matlab R2017 (Mathworks).

### Computational setup

The interactions between RONS and cysteine were investigated with reactive molecular dynamics (MD) simulations, using the density-functional based tight-binding (DFTB) method. Second order DFTB (*i.e*. SCC-DFTB complemented with the mio parameter set) was used, which employed the second series expansion of the total Kohn-Sham energy, as used in conventional density functional theory (DFT) calculations^69^. The result was a self-consistent field algorithm considering the charges of the atoms. These calculations are, however, very time-consuming, which greatly limits the time scale that can be calculated (*i.e*., ps-scale). Because of this, only the fast events can be simulated when using DFTB. More information can be found in the work of Elstner *et al*.^70^. Prior to the MD impact simulations, a cysteine molecule was introduced in a cube with an edge length of 20 Å. The cube was filled with equilibrated water molecules. To eliminate any stresses resulting from introducing the biochemical structure in the solution, the system was further equilibrated at room temperature for 10 ps using a canonical ensemble (temperature and volume were kept constant). A single reactive species was introduced per simulation by replacing a water molecule with the reactive species (minimum distance of 5 Å to the molecule to avoid initial interactions). All impact simulations were performed at room temperature using a Berendsen thermostat with a coupling constant of 100 fs and applying periodic boundary conditions. All simulations were performed using a time step for integration of 0.25 fs for a total time scale of 10 ps. Every interaction was simulated using 50 independent runs. It should be noted that these are only general indications about the simulated results and should not be directly linked to experimental data. The simulated statistics are influenced by the number of calculations performed and by the fact that only fast events can be considered (observed within 10 ps). All statistics, based on the total number of performed simulations, can be found in the supplementary information.

## Electronic supplementary material

Supplementary Information

Supplementary Dataset 1
