# Supplementary material for: Chemical fingerprints of cold physical plasmas – an experimental and computational study using cysteine as tracer compound
Source: Sci Rep. 2018 May 16;8:7736. doi: 10.1038/s41598-018-25937-0 (PMC5955931; doi:10.1038/s41598-018-25937-0)
Supplement: Supplementary file 1 — Supplementary Information [file 41598_2018_25937_MOESM1_ESM.docx]

Chemical fingerprints of cold physical plasmas – an experimental and computational study using cysteine as tracer compound

**J.-W. Lackmann*, K. Wende*, C. Verlackt, J. Golda, J. Volzke, F. Kogelheide, J. Held, S. Bekeschus, A Bogaerts, V. Schulz-von der Gathen, K. Stapelmann**

Supplementary information

Supporting Information Figure 1

**Figure S1**: Measurements of nitrite, nitrate, and hydrogen peroxide for COST-Jet (black) and kinpen (grey). Treatment times were 10 min for all samples, all other conditions as described for cysteine experiments. All experiments were performed in duplicates (both shown) for each condition.

**Experimental for species determination**

The detection of nitrite (NO_2_^-^) and nitrate (NO_3_^-^) was performed by ion chromatography (ICS-5000, Thermo). After treatment, samples were diluted three fold using MS-grade water (Chemsolv). Ten (10) µL were injected onto a system consisting of an IonPac guard column and an IonPac AS23 anion exchange column (2 x 250 mm, both Thermo Fisher Scientic). An isocratic mobile phase (4.5 mM Na_2_CO_3_/0.8 mM NaHCO_3_) with a flow of 250 µl/min was used. Beside conductivity, data were collected from a UV detector setup (210 nm). The system was calibrated using the Dionex 7-anions standard (Thermo Scientific) on a weekly basis. Each individual sample was injected twice and two independent experiments were performed for each condition.

**Colorimetric assay**

Hydrogen peroxide (H_2_O_2_) was detected via the colorimetric reaction with xylenol orange using a commercially available assay (Pierce™ Quantitative Peroxide Assay Kit, Thermo Scientific) according to the manufacturer’s protocol. The dye formed by the reaction was quantified using a Tecan Infinite M200 Pro plate reader (Tecan) at 595 nm. Each 96 well plate contained a standard curve (0 to 150 µM, in triplicates) and the samples. Each individual sample was measured in triplicates and two independent experiments were performed.

*SI-Table 1. Summary of the reactions observed in the simulations, as well as the relative occurrence (in percent) at which they were observed. These relative occurrences only present some indication due to the limited statistics that could be applied, inherent to this type of simulations, which are very time-consuming. The values refer to the number of observed reaction products with respect to the number of simulations performed per species and reactants. In cases where the sum of the relative occurrences does not reach 100%, the remaining percentages refer to simulations where no reactions are observed (e.g. 95% in the case of the interaction between reactants 2 and O_2_).*

| **Reactants** | **Species** | **Reaction product** | **Rate (%)** |
| --- | --- | --- | --- |
| 1 | OH | 5 | 38 |
| 1 | OH | 6 | 22 |
| 1 | OH | 3 + 4 | 20 |
| 1 | NO | 2 | 26 |
|  |  |  |  |
| 2 | O_2_ | 7 | 5 |
|  |  |  |  |
| 4 | O_2_ | 20 | 16 |
| 4 | O_3_ | 20 | 16 |
|  |  |  |  |
| 5 | OH | 11 | 80 |
| 5 | O_3_ | 8 | 60 |
| 5 | O_3_ | 9 | 18 |
| 5 | O_2_ | 11 | 5 |
|  |  |  |  |
| 6 | OH | 11 | 84 |
| 6 | NO | 7 | 26 |
| 6 | O_3_ | 9 | 10 |
| 6 | O_2_ | 13 | 6 |
| 6 | O_2_ | 11 | 4 |
| 6 | Cys-(-H) | 21 | 16 |
|  |  |  |  |
| 9 | OH | 10 | 40 |
| 9 | NO | 12 | 60 |
| 9 | O_2_ | 10 | 4 |
|  |  |  |  |
| 11 | OH | 18 | 4 |
| 11 | OH | 9 | 56 |
|  |  |  |  |
| 13 | OH | 15 | 56 |
| 13 | NO | 14 | 24 |
|  |  |  |  |
| 14 | O_2_ | 22 | 4 |
|  |  |  |  |
| 15 | O_2_ | 16 | 8 |
|  |  |  |  |
| 18 | OH | 10 | 80 |
| 18 | O_2_ | 10 | 5 |

*SI-Table 2 – Mass spectrometry data sets*

Please inquire from the authors – [kristian.wende@inp-greifswald](mailto:kristian.wende@inp-greifswald) or [jan-wilm.lackmann@inp-greifswald.de](mailto:jan-wilm.lackmann@inp-greifswald.de) (xlsx-file, 1Mbyte), or download the file SI_MS_Data sets.xlsx from the journals website.
